# Effects of Qutanhuoxue Decoction on AQP7 and AQP9 Expression in Nonalcoholic Fatty Liver Model Rats

**DOI:** 10.1155/2019/5709626

**Published:** 2019-06-02

**Authors:** Ding Zheng, Mengyun Peng, Xiaoning Zhu, Jing Wang

**Affiliations:** Affiliated Traditional Chinese Medicine Hospital of Southwest Medical University, Luzhou 646000, China

## Abstract

The purpose of this study was to investigate the effect of Qutanhuoxue decoction on AQP7 and AQP9 expression in nonalcoholic fatty liver model rats. Nighty male SD rats (six weeks old, 250 ± 10 g) were randomly divided into 5 groups: normal diet group (ND group), high-fat diet (HFD group), HFD + low dose Qd group, HFD + middle dose Qd group, and HFD + high dose Qd group. Rats in ND group were fed with a regular diet, while rats in other groups were fed with high-fat diet. After the success of the molding, HFD + low dose Qd group, HFD + middle dose Qd group, and HFD + high dose Qd group were, respectively, gavaged by Qutanhuoxue decoction with concentration of 4.5g.kg^−1^.d^−1^, 9.0g.kg^−1^.d^−1^, and 18g.kg^−1^.d^−1^. The ND group and HFD group were gavaged by the same volume of physiological saline lavage, once a day. During the period of gavaging, the other four groups continue to be fed with high-fat fodder except ND group. All rats were killed at 14d, 21d, and 28d, respectively. HE staining was used to observe the pathological changes of liver tissues and serum level of ALT AST GGT and TC TG was detected by automatic analyzer. The expression levels of liver AQP9 mRNA and adipose tissue AQP7 mRNA were detected by real-time PCR. Quhuoxue decoction can significantly reduce the liver function (ALT, AST, and GGT) and blood fat (TG, TC) levels of NAFLD rats and reduce the degree of liver fat degeneration. The effect was the best in the HFD + high dose Qd group of 28d. Qutanhuoxue decoction can decrease the expression of liver AQP9 mRNA and increase the expression of adipose tissue AQP7 mRNA. In conclusion, Qutanhuoxue decoction can reduce the degree of hepatic steatosis, which may be closely related to the increase of AQP7 expression in adipose tissue and the decrease of AQP9 expression in liver.

## 1. Introduction

Nonalcoholic fatty liver disease (NAFLD) is a kind of clinical pathological syndrome characterized by TG accumulation in liver cells, which excludes the history of excessive drinking and definite liver damage factors. The disease is insidious and progress is slow [1, 2]. The incidence of NAFLD increases year by year, and it has become the main cause of chronic liver disease in many regions of the world [3]. Effective intervention in NAFLD can reduce the incidence of hepatitis, liver cirrhosis, and liver cancer [4]. Except for symptomatic treatment of western medicine, there is still no specific drug for NAFLD. Chinese medicine has rich clinical experience in treatment of NAFLD, but lack of enough experimental evidence. So further research of traditional Chinese medicine has important clinical significance.

The pathogenesis of NAFLD is very complex. At present, it is generally recognized as the theory of “two hits”. The first hit was the accumulation of TG in the liver. Blocking the accumulation of TG in the liver can avoid the damage to the liver caused by the second hit. A large number of experimental studies have proved that the mobilization and transport of fat is related to aquaporins, especially to AQP7 in fat cells and AQP9 in liver cells [5, 6].

Abnormal expression of AQP7 in fat cells and AQP9 in hepatocytes leads to lipid metabolism disorder when glycerol is out of balance in and out of hepatocytes, and it results in the accumulation of triglycerides in hepatocytes. For example, the deficiency or decrease of AQP7 results in the decrease of glycerol release from fat cells into the blood. The accumulation of glycerol in fat cells leads to increased triglyceride synthesis, which leads to obesity. The increased expression of AQP9 will lead to a large amount of glycerol entering the liver cells, exceeding the liver's compensatory capacity, thus leading to the increase of liver triglyceride synthesis.

The coordination and regulation of AQP7 and AQP9 play an important role in the control of obesity and hepatic steatosis. Leire Méndez-Giménez found that sleeve gastrectomy, a widely applied bariatric surgery procedure, restores the coordinated regulation of fat-specific AQP7 and liver-specific AQP9, thereby improving whole-body adiposity and hepatic steatosis [7]. AQP3 and AQP7 may facilitate glycerol efflux from adipose tissue while reducing the glycerol influx into hepatocytes via AQP9 to prevent the excessive lipid accumulation and the subsequent aggravation of hyperglycemia in human obesity [8].

Qutanhuoxue decoction is a clinical proved recipe for NAFLD treatment by sun tongjiao, a professor of traditional Chinese medicine. Previous studies have found that Qutanhuoxue decoction can effectively inhibit the expression of NF-*κ*b and reduce the expression of AQP9 in liver tissue and reduce the accumulation of glycerol in liver cells [9–11]. Therefore, on the basis of previous studies, this study further observed the regulatory effects of Qutanhuoxue decoction on the expression of AQP7 in adipose tissue and AQP9 in liver tissue.

## 2. Materials and Methods

### 2.1. Experimental Animals

Nighty male Sprague-Dawley rats, weighing 250 ± 10g (specific pathogen free, certificate No. SYXK013-065), were purchased from Southwest Medical University laboratory animal center. High-fat feed including 76% basic feed, 1% cholesterol, 10% lard, 1% bile salt, and 2% white sugar were purchased from Chengdu datus laboratory animal company. The experiment has been approved by the ethics committee of the hospital of traditional Chinese medicine affiliated to Southwest Medical University.

### 2.2. Experimental Medicine

Qutanhuoxue decoction was purchased from preparation center of Chinese Medicine Hospital affiliated to Southwest Medical University. The dose conversion is calculated according to the conversion ratio of surface area between humans and animals. The concentrations of TCM in the HFD + low dose Qd group, the middle dose group, and the HFD + high dose Qd group were 4.5g/100ml, 9.0g/100ml, and 18 g/100ml, respectively. The composition of Qutanhuoxue decoction is shown in Table 1.

### 2.3. Experimental Reagent and Apparatus

ALT, AST, and GGT kit were acquired from Chengdu Borek Biotechnology Co. Ltd. (Batch Numbers D1610143, D1612083, and D1611053), TC and TG kit were acquired from Wenzhou Eastern Europe Biological Engineering Co. Ltd. (Batch Numbers 160513 and 151104), and reverse transcription kit and real-time PCR kit were acquired from Chengdu Borek Biotechnology Co. Ltd. The instruments used were as follows: AU600 fully automatic biochemistry analyzer (Olympus Company, Japan); Spectrophotometer type UV-752 (Shanghai, China); PCR amplifiers and gel scanning imaging systems (BIO-RAD Company, USA).

### 2.4. Grouping and Modeling

One week after adaptive feeding, ninety male SD rats are randomly divided into 5 groups: ND group, HFD group, HFD + low dose Qd group, HFD + middle dose Qd group, and HFD + high dose Qd group. Rats in ND group were fed with a regular diet, while rats in the other four groups continue to be fed high-fat fodder. After the success of the molding, HFD+low, HFD+middle, and HFD+ high dose Qd groups were, respectively, gavaged by Qutanhuoxue decoction 4.5g.kg^−1^.d^−1^, 9.0g.kg^−1^.d^−1^, and 18g.kg^−1^.d^−1^. The ND group and HFD group were gavaged by the same volume of physiological saline lavage, once a day. During the period of gavaging, the other four groups continued to be fed with high-fat fodder except ND group. All rats were killed at 14d, 21d, and 28d, respectively.

The rats were anesthetized by intraperitoneal injection of 1% pentobarbital (4ml/kg, which can be added according to the actual situation in the experiment). After the rats were anesthetized, they were laid on their backs and their limbs were quickly fixed on the operating board. After disinfection of the skin preparation, incision was made along the midline of the abdomen to fully expose the inferior vena cava. In pursuance of 4 ml, vacuum blood collection needle placed at room temperature after 20 min 1370 g centrifugal 8 min, take supernatant, EP tube packing with new enzymes in - 80°C refrigerator for measurement. After blood collection, the liver was fully exposed and its size and color were observed. 1‰DEPC water treated blade was used to take liver tissue with moderate thickness and put it into 10% formalin for fixation, and then 100 mg liver and peripheral adipose tissue of epididymis were taken into cryopreservation tube, respectively, and number them. Finally putting them into liquid nitrogen quickens frozen vacuum flask, and they were transferred to - 80°C cryogenic refrigerator after taking the specimens.

### 2.5. Histopathology Evaluation

The liver specimens were selected from all rats about 1 cm from the edge of the maximum hepatic lobule. The liver issue was fixed with formaldehyde solution for 24 h. Then they were embedded in paraffin and sliced at 5 *μ*m thickness. And they were stained with hematoxylin and eosin (HE) and then observed under optical microscope for the pathomorphological changes. Steatosis scoring criteria are shown in Table 2.

### 2.6. Biochemical Detection

The concentration of total cholesterol (TC), triglyceride (TG), alanine aminotransferase (ALT), aspartate transaminase (AST), and *γ*-glutamyl transpeptidase (GGT) in the serum were measured by an automatic analyzer.

### 2.7. Real-Time PCR

Total RNA was extracted with RNA TRIzol™ reagent, according to the manufacturer's specifications of the reagent. The concentration and purity were measured with an ultraviolet spectrophotometer. Reverse transcription was performed according to the requirements of the kit. Reverse transcription reaction condition was 42°C 20 min, 99°C 5 min, and 4°C 5 min. After the reverse transcription reaction was finished, the samples were centrifuged instantaneously and stored at −20°C for use. Polymerase Chain Reaction was performed according to following protocols: denaturation was carried out at 95°C for 30s first. Each cycle of amplification consisted of 5s of denaturation at 95°C, followed by 10s of annealing (54°C) and 15s of extension (72°C) steps. The mRNA numbers of aqp9, aqp7 and GADPH of SD rats were inquired, and the primers designed by the company are shown in Table 3.

### 2.8. Statistical Analysis

All data were analyzed using SPSS 23.0 software. Values in the text are means ± standard deviation $\left(\overset{-}{x}\pm s\right)$. Statistical analyses were performed using analysis of variance (ANOVA) followed by the least significant difference (LSD). Values of P <0.05 were considered significant.

## 3. Result

### 3.1. The Effect of Qutanhuoxue Decoction on the Growth Rate of Rats Body Weight

**Figure 1:**
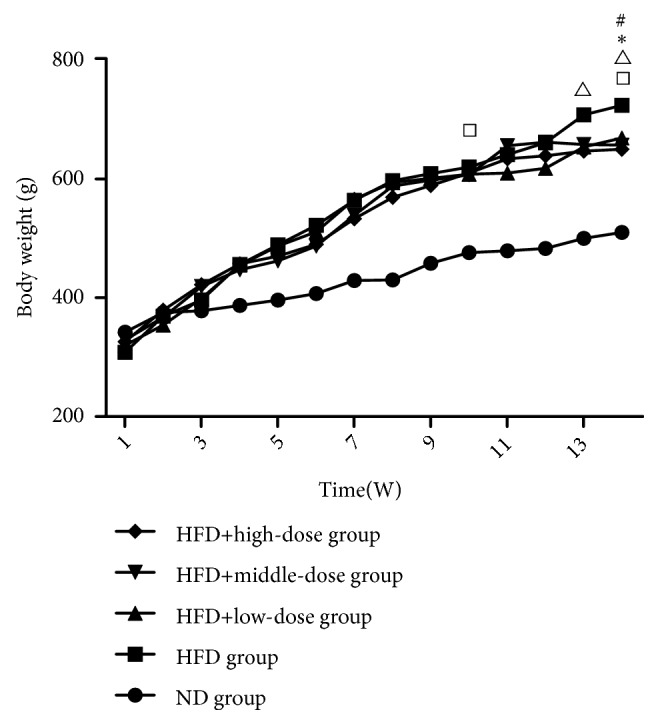
The effect of Qutanhuoxue decoction on the growth rate of rats body weight. Body weight curse ^#^*P<0.05* HFD group versus HFD + high dose Qd group, *∗P<0.05* HFD group versus HFD + middle dose Qd group, ^△^*P<0.05* HFD group versus HFD + low dose Qd group, and ^□^*P<0.05* HFD group versus ND group.

As shown in Figure 1, the weight curve for the ND group was lower than those of other four groups except at 1w (*P<0.05*). At 10w, there was no significant difference in weight of the rats in the other groups except for ND group (*P>0.05*). At 14w, the weight of high dose group was higher than those of the other four groups (*P<0.05*). These results indicated that Qutanhuoxue decoction can reduce the growth rate of rats body weight.

### 3.2. The Effect of Qutanhuoxue Decoction on Hepatic Steatosis of NAFLD Rats

**Figure 2:**
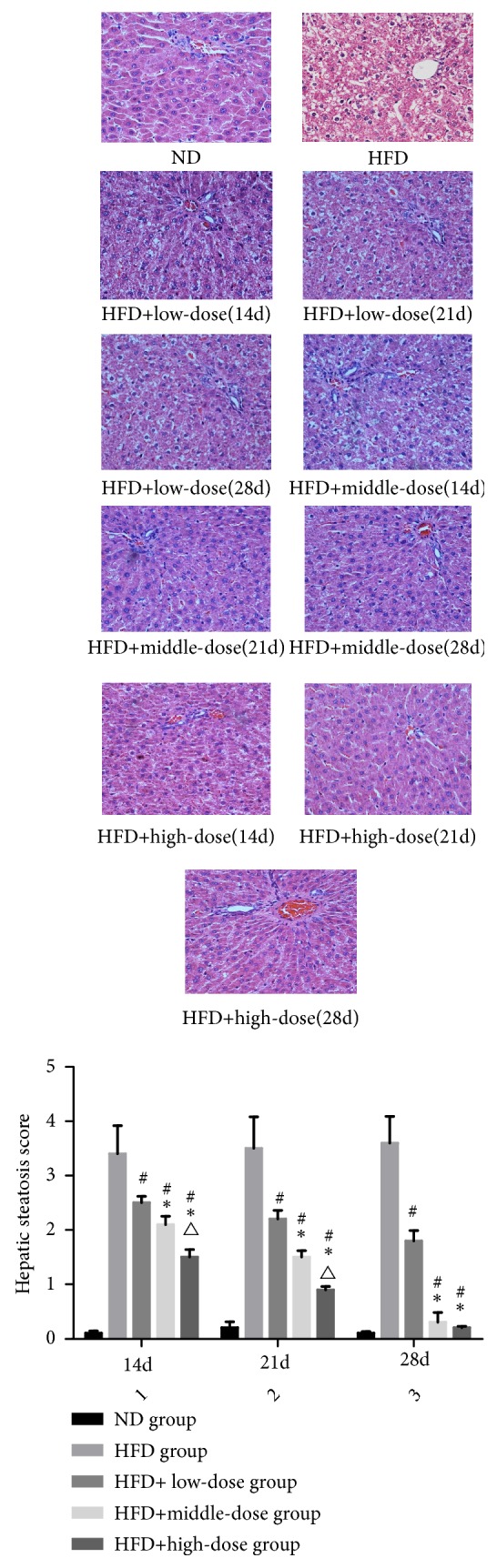
The effect of Qutanhuoxue decoction on hepatic steatosis of NAFLD rats. ^#^*P<0.05* compared with HFD group versus HFD + high dose Qd group, *∗P<0.05* compared with HFD + low dose Qd group, and ^△^*P<0.05* compared with HFD + middle dose Qd group.

As shown in Figure 2, the hepatic lobule of ND group rats was intact in structure and no fat degeneration and necrosis were observed in cells. In the HFD group, the hepatic lobule structure of rats was seriously damaged, and the hepatocytes were severely diffuse with steatosis. The fatty liver degeneration of the low, middle, and high dose group was improved inordinately after 14d, 21d, and 28d treatment. At 14d and 21d, the liver steatosis score of the HFD + high dose Qd group was lower significantly than that of the HFD + middle dose Qd group* (P<0.05)*. At 28d, the liver steatosis score of the HFD + high dose Qd group and middle dose group was lower significantly than that of the lower-dose group* (P<0.05). *There was no significant difference between the middle and HFD + high dose Qd groups* (P>0.05)*.

### 3.3. The Effect of Qutanhuoxue Decoction on Serum Levels of ALT, AST, and GGT

**Figure 3:**
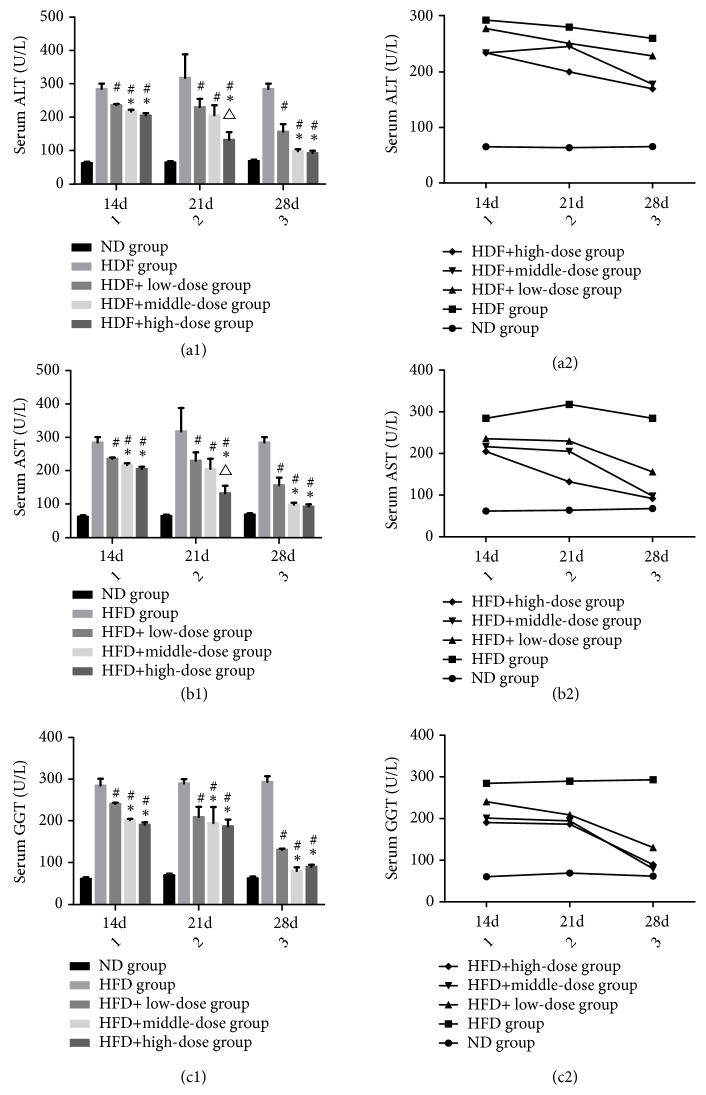
The effect of Qutanhuoxue decoction on serum levels of ALT, AST, and* GGT. (a1, a2) serum ALT. (b1, b2) serum AST. (c1, c2) serum GGT. *^#^*P<0.05 compared with HFD group versus HFD + high dose Qd group, ∗P<0.05 compared with HFD + low dose Qd group, and*^△^*P<0.05 compared with HFD + middle dose Qd group.*

As shown in Figure 3, compared with the ND group, the serum levels of ALT, AST, and GGT in HFD group were significantly increased. Compared with the HFD group, serum levels of ALT, AST, and GGT in low, middle, and high dose groups decreased to varying degrees. The overall analysis found that there was a significant difference between the drug concentration and time levels* (P<0.05)*, and there was no interaction between the two factors. The serum level of AST, ALT, and GGT at 28d was lower than that at 14d and 21d* (P<0.05)*. At 28d, The decline of serum level of AST, ALT, and GGT in the middle and high dose group was more obvious than that in the low dose group* (P<0.05)*, and there was no significant difference between the middle and HFD + high dose Qd groups.

### 3.4. The Effect of Qutanhuoxue Decoction on Serum Levels of TC and TG

**Figure 4:**
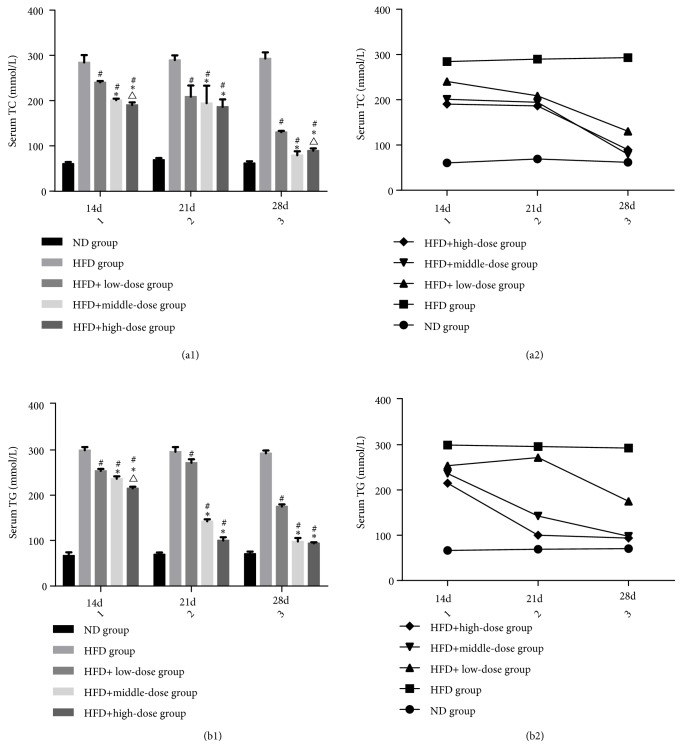
The effect of Qutanhuoxue decoction on serum levels of TC and TG.* (a1, a2) serum TC. (b1, b2) serum TG. *^#^*P<0.05* compared with HFD group versus HFD + high dose Qd group, *∗P<0.05* compared with HFD + low dose Qd group, and ^△^*P<0.05* compared with HFD + middle dose Qd group.

As shown in Figure 4, compared with the ND group, serum levels of TC and TG in HFD group were significantly increased. Compared with the HFD group, serum levels of TC and TG in low, middle, and high dose groups decreased to varying degrees. The serum level of TC and TG at 28d was lower than that at 14d and 21d in low, middle, and high dose groups* (P<0.05)*. At 28d, The decline of serum level of TC and TG in the middle and high dose group was more obvious than that in the low dose group* (P<0.05)*, and there was no significant difference between the middle and HFD + high dose Qd groups.

### 3.5. The Effect of Qutanhuoxue Decoction on aqp7 and aqp9 Expression

**Figure 5:**
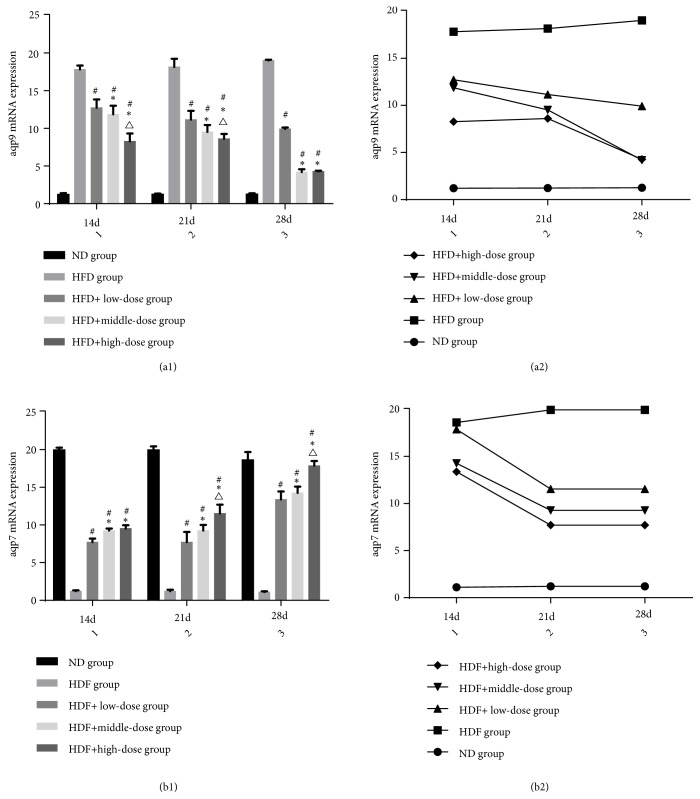
The effect of Qutanhuoxue decoction on aqp7 and aqp9 expression.* (a1, a2) aqp9 mRNA expression, (b1, b2) aqp7 mRNA expression. *^#^*P<0.05* compared with HFD group versus HFD + high dose Qd group, *∗P<0.05* compared with HFD + low dose Qd group, and ^△^*P<0.05* compared with HFD + middle dose Qd group.

As shown in Figure 5, compared with the ND group, the expression of liver aqp9 mRNA in HFD group was significantly increased and the expression of adipose tissue aqp7 mRNA in HFD group was significantly decreased* (P<0.05)*. Compared with the HFD group, the expression of liver aqp9 mRNA in low, middle, and high dose groups decreased and the expression of adipose tissue aqp7 mRNA in low, middle, and high dose groups increased to varying degrees* (P<0.05)*. At 28d, the expression of aqp9 mRNA was lower than that at 14d and 21d and the expression of adipose tissue aqp7 mRNA was higher than that at 14d and 21d* (P<0.05)*. These results indicated that Qutanhuoxu granule can decrease the expression of liver aqp9 mRNA and increase the expression of adipose tissue aqp7 mRNA.

## 4. Discussion

Nonalcoholic fatty liver disease (NAFLD) is closely related to heredity, environment, diet, and obesity [12]. NFLD is the liver manifestation of metabolic syndrome, with a high incidence rate worldwide [13]. At present, NAFLD has become the main cause for hepatocellular carcinoma in the United States [14, 15]. There are still no effective therapies for NAFLD patients in western countries [16].

In this study, high-fat induction NAFLD rat model was adopted. Ultrasound is the most commonly used method for NAFLD detection in clinical practice, but liver biopsy is the gold standard for NAFLD diagnosis [17]. In this study, HE staining showed severe fatty degeneration in the liver of the HFD group, indicating that the model was successfully established. The results of this study indicated that Qutanhuoxue decoction could significantly improve hepatic steatosis and reduce level of serum TC, TG, ALT, and AST, consistent with previous studies.

Studies have shown that the mobilization, transportation, and liver reuptake of fat are correlated with the water-glycerol channel protein [5, 6]. AQP7 and AQP9 played a key role in the first hit. AQP7 is widely distributed in human white fat and brown fat [18]. In fat cells, the AQP7 are mainly distributed around the nucleus. When TG decompose, the tissue adrenaline release increases and then promotes AQP7 by AC-cAMP-PKA way to the membrane transfer from around the nucleus, forming across the membrane of the four polymers to mediated glycerol transport out of the cell [19]. AQP9 is a glycoprotein polypeptide chain, which is on the cell membrane by homologous four polymers, can guarantee the stability of the AQP9 and the distribution of normal function [20]. AQP9 is the most abundant expression in the liver and also expressed in other organs. According to its expression intensity, it is expressed in the liver, testicles, brain, and lungs [21, 22].

The experimental results showed that Qutanhuoxue decoction could promote the expression of fat cells aqp7 and reduce the expression of liver cell aqp9. When the expression of aqp7 increased, the transport of glycerol in adipocytes increased and the synthesis of triglycerides in adipocytes decreased. When the expression of aqp9 decreased, the amount of glycerol transported to the liver decreased, so the amount of glycerol and fatty acids entering the liver decreased, and the liver synthesized triglycerides decreased.

When aqp7 expression increased and aqp9 expression decreased, the amount of glycerol released into the blood increased and absorbed into the liver decreased. So the level of glycerol in the blood increased. But the levels of triglycerides in the blood in this experient droped. It illustrated that the glycerin in the blood may not participate in the formation of TG. So further studies are needed on the metabolic processes of glycerol. There was no evidence that aquaporins are directly related to serum cholesterol, but cholesterol synthesis is regulated by serum insulin, increasing cholesterol production. Qutanhuoxue decoction may improve insulin resistance by restoring the expression of aqp7 and aqp9. Serum insulin will decrease and reduce cholesterol synthesis, but the specific mechanism needs further study.

In order to verify the optimal efficacy of Qutanhuoxue decoction in the treatment of NAFLD with different concentrations and treatment duration. In this experiment, low, middle, and high concentration groups were set up, with treatment duration of 14d, 21d, and 28d, respectively. The results indicated that different concentrations and treatment time had different curative effects in treating NAFLD rats with Qutanhuoxue decoction. Among which the high dose treatment with expectorant and huoxue decoction for 28d group had the best curative effect.

In conclusion, Qutanhuoxue decoction has the effect of treating NAFLD with multiple levels and multiple targets which restoring the balance of glycerol in and out of hepatic cells and alleviating the oxygen stress response. So it could reduce the accumulation of TG in hepatocytes and alleviating hepatocyte injury.

## Figures and Tables

**Table 1:** The composition of Qutanhuoxue decoction.

Chinese name	English name	Proportion (g)
chenpi	TangerinePeel	10
fulin	poria	10
jiangbanxia	rhizoma pinelliae	10
yiyiren	semen coicis	20
zexie	rhizoma alismatis	10
yujin	radix curcumae	15
danshen	radix salviae miltiorrhizae	15
shanzha	fructus crataegi	15
chaihu	radix bupleuri	10
huangqin	radix scutellariae	10
juemingzi	semen cassiae	10

**Table 2:** Liver steatosis scoring criteria.

pathological grading	Number of steatotic hepatocytes/total cells	Steatosis score
0	0	0
I	<1/3	1
II	1/3-2/3	2
III	>2/3	3
IV	≈1	4

**Table 3:** Upper and lower primers of aqp9, aqp7, and GADPH in SD rats.

gene	sequences of the primers	amplified fragments
aqp7	Sense primer: tcgtgactgggatgctgc	158bp
	Reverseprimer: acgggatgggttgattgc	
aqp9	Sense primer: aaggacggtgccaagaa	197bp
	Reverseprimer: atcacgactgccgatgc	
GAPDH	Sense primer: ccatccacagtcttctgagt	141bp
	Reverse primer: cctcaagattgtcagcaat	

## Data Availability

The data used to support the findings of this study are available from the corresponding author upon request.

## Supplementary Materials

Supplementary Materials Supplemental Table 1. The effect of Qutanhuoxue decoction on serum levels of ALT, AST, and GGT. Supplemental Table 2. The effect of Qutanhuoxue decoction on serum levels of TC and TG. Supplemental Table 3. The effect of Qutanhuoxue decoction on serum levels of GGT. Supplemental Table 3. The effect of Qutanhuoxue decoction on serum levels of AQP-7 and AQP-9.
