# Supplementary material for: Effects of Qutanhuoxue Decoction on AQP7 and AQP9 Expression in Nonalcoholic Fatty Liver Model Rats
Source: Evid Based Complement Alternat Med. 2019 Jun 2;2019:5709626. doi: 10.1155/2019/5709626 (PMC6582829; doi:10.1155/2019/5709626)
Supplement: Supplementary Materials — Supplemental Table 1. The effect of Qutanhuoxue decoction on serum levels of ALT, AST, and GGT. Supplemental Table 2. The effect of Qutanhuoxue decoction on serum levels of TC and TG. Supplemental Table 3. The effect of Qutanhuoxue decoction on serum levels of GGT. Supplemental Table 3. The effect of Qutanhuoxue decoction on serum levels of AQP-7 and AQP-9. [file 5709626.f1.pdf]

Supplementary Materials

Supplemental Table 1: The effect of Qutanhuoxue decoction on serum levels of ALT, AST and GGT

group	ALT			AST		
	14d	21d	28d	14d	21d	28d
Normol group	65.31±1.95	63.64±7.35	65.64±7.16	61.98±4.073	63.98±4.123	67.98±4.163
Model group	293.29±9.52#	280.60±7.35#	260.29±14.14#	284.40±16.34#	317.74±70.28#	284.40±16.34#
Low-dose group	278.15±7.49*	251.48±7.69*	228.82±4.01*●	235.53±3.71*	229.98±24.71*	155.98±23.13*●
Middle-dose group	234.15±6.04*	245.93±8.66*	177.95±4.04*	216.42±5.82*	205.31±30.14*	97.76±5.67*
High-dose group	234.48±5.00*●	200.15±12.15*●	169.71±6.01*	204.86±7.21*●	132.08±22.84*●	91.76±7.313*

*Table 1:#P＜0.05 compared with normol group. *P＜0.05 compared with model group,•P＜0.05 compared with High-dose group.*

Supplemental Table 2: The effect of Qutanhuoxue decoction on serum levels of TC and TG

group	TC			TG		
	14d	21d	28d	14d	21d	28d
Normol group	88.71±7.12	80.71±4.84	81.71±6.14	66.66±7.29	69.33±4.28	70.66±4.87
Model group	284.90±14.56#	263.60±6.00#	269.90±9. 51#	299.45±7.14#	296.12±10.24#	292.78±6.09#
Low-dose group	238.93±6.667*	242.71±7.6694*	193.82±9.82*●	253.80±4.30*●	271.90±7.57*●	175.23±4.64*●
Middle-dose group	225.38±2.34*	210.04±14.38*	119.37±15.90*●	236.66±5.06*●	142.57±4.31*●	97.85±7.72*
High-dose group	194.93±17.90*●	202.93±5.69*●	105.71±7.16*	215.00±3.97*●	100.00±7.03*	93.80±2.50*

Table 2:#P＜0.05 compared with normol group. *P＜0.05 compared with model group, •P＜0.05 compared with High-dose group.

Supplemental Table 3: The effect of Qutanhuoxue decoction on serum levels of GGT

group	GGT		
	14d	21d	28d
Normol group	60.51±4.073	69.18±4.123	61.84±4.163
Model group	284.40±16.34#	289.74±10.28#	293.32±13.354#
Low-dose group	240.51±3.006*	208.73±24.882*	130.51±2.775*●
Middle-dose group	201.09±3.251*	194.66±38.445*	79.62±8.572*
High-dose group	190.51±5.748*●	186.51±16.252*●	89.40±5.033*

Table 3:#P＜0.05 compared with normol group. *P＜0.05 compared with model group, ●P＜0.05 compared with High-dose group.

Supplemental Table 4: The effect of Qutanhuoxue decoction on serum levels of AQP-7 and AQP-9

group	$2^{-\Delta\Delta Ct}$ （AQP9 ）			$2^{-\Delta\Delta Ct}$ （AQP7）		
	14d	21d	28d	14d	21d	28d
Normol group	1. 21±0. 19	1. 23±0. 11	1. 25±0. 13	1. 10±0. 10	1. 21±0. 19	1. 21±0. 11
Model group	17. 82±0. 52#	18. 16±1. 08#	19. 02±0. 09#	18. 61±1. 06#	19. 94±0. 49#	19. 60±0. 33#
Low-dose group	12. 73±1. 12*	11. 16±1. 16*	9. 93±0. 19*●	17. 87±0. 62*	11. 54±1. 15*	9. 87±0. 43*●
Middle-dose group	11. 87±1. 15*	9. 53±0. 92*	4. 20±0. 38*	14. 27±0. 83*	9. 27±0. 75*	3. 27±0. 26*
High-dose group	8. 28±1. 04*●	8. 61±0. 66*●	4. 28±0. 10*	13. 38±1. 06*●	7. 71±1. 36*●	3. 71±0. 49*

Table 4:#P＜0.05 compared with normol group. *P＜0.05 compared with model group, ●P＜0.05 compared with High-dose group.
